# Computer Simulation of Leadership, Consensus Decision Making and Collective Behaviour in Humans

**DOI:** 10.1371/journal.pone.0080680

**Published:** 2014-01-17

**Authors:** Song Wu, Quanbin Sun

**Affiliations:** School of Built and Environment, University of Salford, Salford, United Kingdom; Koc University, Turkey

## Abstract

The aim of this study is to evaluate the reliability of a crowd simulation model developed by the authors by reproducing Dyer et al.'s experiments (published in *Philosophical Transactions* in 2009) on human leadership and consensus decision making in a computer-based environment. The theoretical crowd model of the simulation environment is presented, and its results are compared and analysed against Dyer et al.'s original experiments. It is concluded that the simulation results are largely consistent with the experiments, which demonstrates the reliability of the crowd model. Furthermore, the simulation data also reveals several additional new findings, namely: 1) the phenomena of sacrificing accuracy to reach a quicker consensus decision found in ants colonies was also discovered in the simulation; 2) the ability of reaching consensus in groups has a direct impact on the time and accuracy of arriving at the target position; 3) the positions of the informed individuals or leaders in the crowd could have significant impact on the overall crowd movement; and 4) the simulation also confirmed Dyer et al.'s anecdotal evidence of the proportion of the leadership in large crowds and its effect on crowd movement. The potential applications of these findings are highlighted in the final discussion of this paper.

## Introduction

Collective movement and consensus decision making have been found in many animal groups, such as honey bees [1]–[3], fishes [4]–[6], and monkeys [7]. Experiments to investigate why human groups make consensus decisions and how the informed individuals influence the group's movement are limited. For example, Dyer et al. have performed a series of experiments [8], [9] on consensus decision making on human groups. Their studies showed similar findings to animal groups, such as the minority can lead the group effectively and the importance of the positions of the informed individuals in small size human groups. But the findings on large size groups were described as anecdotal due to “the logistical difficulties” (insufficient experiment samples). *(In the following sections, the word “experiments” refers to the experiments in Dyer et al.'s study (2009), if no explicit reference has been made)*.

To overcome such “logistical difficulties” for larger groups, one possible solution is to employ crowd simulation technology which utilises a computer programme to simulate crowd behaviour. Up till now, many studies have used the approach on both animal groups and pedestrians' movement. For example, Couzin et al. [10] presented a model to interpret how the informed individuals could influence others in the group to reach a consensus decision. A number of models [11] have been developed to represent some typical crowd phenomena (e.g. clogging, pushing, unadventurous exiting and faster-is-slower) and other crowd models [12]–[16] have been developed to simulate the counter-flow of crowd movement.

In the past two decades, there has been significant development on crowd models. While the early crowd models considered the crowd and its mechanism as a whole, the more recent ones modelled group behaviour on an individual basis, which has become popular in modern crowd modelling research. Thus, modern crowd models can be divided into three categories based on how they interpret and process individual behaviours: force-based models, Cellular Automata (CA) models, and agent-based models.

Force-based models consider individuals in the crowd are affected by some forms of forces (though not to be confused or regarded as the same forces in physics). In force-based models, the motions of individuals are determined by the total effects of forces. Forces are calculated through a set of formulas, which represent behaviours. This idea was first introduced by Reynolds [17] in the ‘Boids’ program which was developed to simulate the motion of bird flocks. In the flock, each bird updated its position by applying a steering force. Later, Helbing and Molnar [18] proposed the Social Force model to describe the movement of pedestrians. In this model, the pedestrian's movement is determined by the forces that are generated from his/her own desire and repulsions/attractions from other pedestrians and objects. Helbing et al. [19] further developed this model to simulate panic situations by considering social psychology. Parisi and Dorso [20] introduced the degree of panic into the Social Force model to simulate the “faster is slower” effect when exiting a room. The force-based approach has its limitation as individual decisions are often ignored in these models. This is because the process of thinking and decision making is difficult to represent only by mathematical equations.

CA models divide the environment/building into small equal sized cells (which can only be occupied by one person at one time) and focus on the state-changing rules, which decide how a person moves to an empty adjacent cell. Wolfram [21] first introduced the way to use cellular automata to model crowds. Since then, CA models have been employed to study the behaviours of pedestrians [22], [23] and indoor crowds [24], [25]. Although CA models are fast and easy to implement, they suffer the limitations of fixed crowd density and unrealistic flow rates through doors due to its nature of fixed cell size [26] (CA models cannot represent the difference between a door with 1.5 cells in width and another door with 1 cell in width. In both cases, only one person can get through the door because the door is represented by one cell. In other words, the fixed cell size is not able to align to the actual geometry accurately).

Agent-based models were introduced to integrate human decision making process in crowd simulation. These models contain autonomous agents, which make their own decisions and can be used to simulate human systems [27], [28]. Agent-based models are usually combined with CA models [29] or force-base models [30] to represent the movement of agents. As agents can be easily attributed, individual behaviours have been considered in many agent-based models and the results suggest that individual behaviours could affect crowd behaviours. For example, Braun et al.'s [30] simulation showed that the flow (person per second) of crowds become slower when the dependency level of group members in the crowd increased. Pelechano and Badler [31] found that effective communication and trained leaders in crowds can increase evacuation efficiency. Shendarkar et al. [32] suggested that increasing the number of police during evacuation could reduce the evacuation time to some extent.

However, most of these models could only consider a small number of behaviours. Challenges still remain of how to flexibly integrate multiple human behaviours into a crowd model without significant reconfiguration, e.g. Moussaid et al. [33] proposed a heuristic-based approach to simply the behaviour representation but it lacks integration of crowd heterogeneity. The authors' previous study [34] introduced a concept of a generic crowd model, which combined the force-based model and the agent-based model together through integrating forces into the decision-making process and then translating behaviours into forces to affect the motions of agents. In this paper, this model is applied to Dyer's two experiments to further validate the reliability of the model and to explore further group consensus behaviour through simulation.

In the next part of the paper, the theoretical basis and the mechanisms of the crowd model implemented in the simulations will be described. Secondly, how the simulation was configured to reproduce Dyer's experiments will be revealed. Thirdly, a comparison and analysis of the simulation results with Dyer's original experiments results are presented. Finally, findings and limitations of this research will be concluded.

## Materials and Methods

### Crowd Model

The authors' crowd model is a hybrid model based on the force-based and the agent based modelling approach. It represents the bi-directional effects between entities (i.e. between an individual agent and other objects or between agents) by adopting the concept of forces. The combination of the effects of the forces will determine the behaviours of individuals by taking into account agents' personal attributes. The multi-agent system approach is used to simulate the decision making process of individual agent in a crowd.

The crowd model can be viewed on two levels. In the lower level, the model interprets how an agent changes its position. An agent's movement is determined by the effects of forces generated from itself, nearby crowd and surrounding objects. The agent's position is updated by applying the behaviour effects (can be viewed as the results of the forces), which are calculated through a set of pre-defined behaviour rules (via formulas). The behaviour effect is represented as the displacement of the agent in the update interval *(the theoretical basis of representing behaviour effect as the positional change of the agent is based on kinematics. In kinematics, the displacement of an object in a period of time can be calculated via its average velocity during that period. Therefore, the behaviour effect is viewed as an equivalent to agent's average velocity and the calculations are based on Classical Mechanics and Newtonian laws)*. The Cartesian coordinate system has been employed to represent the precise and continuous position of each agent in the model.

In the higher level, the model describes how an agent reacts to others and how it decides and conducts its own behaviour. A multi-agent system approach is adopted to simulate the decision making process. The agent's behaviours are decided by itself, based on behavioural preferences, agent's status, personal attributes and its perception of the simulation world. As a result, corresponding behaviours will be determined and relevant behaviour rules will be applied to translate them into the behaviourial effects. The combination of those effects will determine the agent's movement at the lower level.

The algorithm of how an agent updates its movement can be summarized as below:

1. The agent selects the appropriate behaviour rules based on the scenario: multiple behaviours can be selected (detailed behaviour decision process for the simulations is presented in “Simulation configuration” section)
2. Calculate the behaviour effect of each selected behaviour rule (the formulas to calculate the behaviour effect are described in the next section)
3. Combine the selected Behaviour Effects (i.e. the Euclidean summation of the behaviour effects of selected behaviour effects).
4. Update the agent's position by applying the combined Behaviour Effect.

#### Formulas for behaviour effects

Formula 1 is the core formula for behaviour effect calculation.
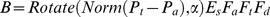
(1)


Formula 1: Core formula - behaviour effect calculation. P_a_ is the position of the agent. P_t_ is the position of the behaviour target. α is the angle for the *Rotate* action (anti-clockwise). *Norm* refers to the standard normalisation operation on a vector. E_s_ is the base movement effect based on the agent's speed in the simulation. F_a_ is the coefficient to represent the influence on behaviour effect from agent itself. F_t_ is the coefficient to reflect the influence on behaviour effect from the target. F_d_ is the coefficient to reflect the influence on behavior effect due to the distance between the agent and its target.

As one agent may have several behaviour effects as the results of the behaviour effects, the agent's position can be updated via the following at the same time, the overall behaviour effect of N number of behaviours is combined by following the rule of addition for Euclidean vector. Because the agent's movement (displacement) is considered formula 2
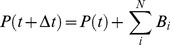
(2)


Formula 2: The formula of agent's position update. *P* represents the agent's position. Δt represents the update interval. ΣB_i_ represents the overall behaviour effect calculation of N behaviours

#### Formulas for specific behaviour rules

In the model, a set of pre-defined behaviour rules have been established *(only the behaviour rules that were used in this simulation have been introduced)*: The calculation of the behaviour rules are all derived from the core formula (Formula 1).

**Seek to:** This behaviour rule describes an agent moves towards the target directly. Its behaviour effect can be calculated using the core formula with the following settings apply:

- The behaviour angle *α* is set to 0 because the agent is moving directly towards the target.
- The parameter F_d_ equals to 1 because the behaviour effect is irrelevant to distance.

The derived formula (3) for the behaviour rule is:
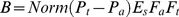
(3)


Formula 3: Seek to target effect calculation. P_a_ is the position of the agent. P_t_ is the position of the behaviour target. *Norm* refers to the standard normalisation operation on a vector. E_s_ is the base movement effect based on the agent's speed in the simulation. F_a_ is the coefficient to represent the influence on behaviour effect from agent itself. F_t_ is the coefficient to reflect the influence on behaviour effect from the target.

**Wandering:** This behaviour rule describes an agent that takes a random route whilst moving. Such a random route is considered as a smooth trajectory rather than a twitchy moving line. Some studies suggested a smooth wandering behaviour can be modelled as the agent changes its moving direction at an angle between [−, +θ] randomly during time Δt [10], [35]. To adapt this approach in this model, the wandering behaviour can be described as the agent seeks to a virtual target in the front and this behaviour has an angle which is randomly chosen from [−, +θ]. Based on the core formula, the following settings can be applied:

- The behavioural angle α is set to a random value in the range of [−, +θ].
- The position of the virtual target P_t_ is a virtual position in the front of the agent's current direction with any distance (distance does not affect the value of the behaviour effect because of the Normalise operation).
- The parameter F_t_ is set to 1 because the target is virtual and should have no effect.
- The parameter F_d_ is set to 1 because the behaviour effect is irrelevant to distance.

The derived formula for the behaviour rule is:
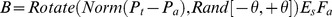
(4)


Formula 4: Wandering effect calculation. P_a_ is the position of the agent. P_t_ is the position of the virtual target in the front. Rand[−,+θ] represents the random selection function of behaviour angle α for the *Rotate* action (anti-clockwise). In the model, by default, θ is set at 0.5 and the probability to change the angle is set at 5% in each update interval (1/60 second) in order to create a smooth wandering trajectory. E_s_ is the base movement effect based on the agent's speed in the simulation. F_a_ is the coefficient to represent the influence on behaviour effect from agent itself.

**Keep in group:** This behaviour rule describes the agent trying to position itself in a group. It includes two effects according to literature: (a) a cohesion effect that moves one to the average position of nearby individuals [17]; (b) an alignment effect that adjusts one's walking direction towards the average heading of nearby individuals [10], [17]. The two behaviour effects can be calculated using the core formula with the following settings:

- The behaviour angle *α* is set to 0 because the agent is moving directly towards the target.
- The position of the virtual target P_t_ is a virtual position which represents the average position of the group.
- The parameter F_t_ is set to 1 because the target is virtual, therefore no effect applied.
- The parameter F_d_ equals to 1 because the behaviour effect is irrelevant to distance.
- In addition to the alignment effect, the behaviour effect calculation has to take the group average orientation into account. The group average orientation is used to adjust the agent's walking direction.

The derived formula (4) for the behaviour rule is:
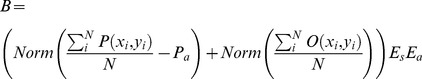
(5)


Formula 5: Behaviour effect calculation for keep in group. P_a_ is the position of the agent. P(xi,yi))is the position of agent_i_ in the group. O(xi,yi) is the orientation of agent_i_ in the group. *(x,y)* represents the position in the Cartesian coordinate system in the simulation. *N* is the total number of the nearby agents which are within the range of the group definition. F_t_ and F_d_ are considered irrelevant to this behaviour and their values are set to 1.

**Repulsive effect from crowd:** This behaviour rule describes the agent receiving an overall repulsive effect from the crowd (the combination of the repulsive effects from everybody) which pushes it away from others in the crowd. Its behaviour effect can be calculated using the core formula with the following settings:

- It is a combination behaviour effect.
- The behaviour angle *α* is set to 180 because the agent is moving away from the target.
- The parameter F_d_ is defined as a piecewise function g(d) given below, which reflects the influence of the distance.
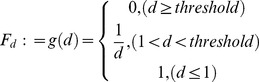
(6)

Formula 6: d represents the distance between the agent self and its nearby agent, where d = ‖pa-pi‖. The unit of d is in pixel. *threshold* represents the distance where the repulsive effect starts to take effect.

The derived formula (7) for the behaviour rule is:
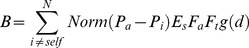
(7)


Formula 7: Repulsive effect from crowd. *N* is the number of the agents in the crowd. P_a_ is the position of agent itself. P_i_ is the position of nearby agent(i). g(d) is the function to adjust the influence of the distance between the two agents on the repulsive effect.

### Dyer et al.'s Original Experiments

In order to evaluate the above crowd model, Dyer et al.'s experiments was selected as they are one of the only few published real-life human experiments available in the field with detailed description and results. The experiments could be described as a group of people walking in a circular arena from the centre to the targets at the periphery by following a set of instructions. The time and accuracy of reaching the target on the periphery are measured as the results.

Two of Dyer et al.'s experiments were selected for simulation. One was in a small arena to test the behaviour of a group of ten, and the other was in a large arena with a group of two hundred participants.

**Figure 1:**
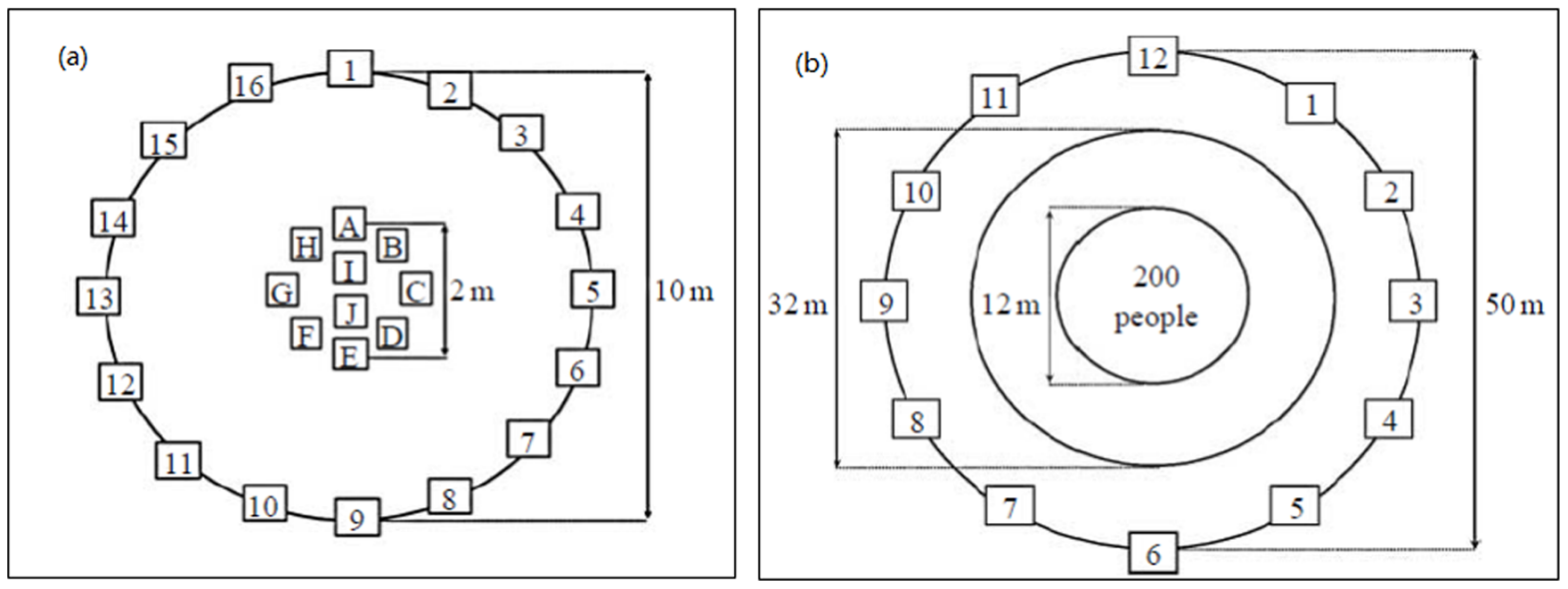
Layout of experiment arenas. (a) small group; (b) large group [9].

For the first experiment, the experiment arena (Figure 1a) was described as “a circular arena with a 10 m diameter that was marked on the floor and cards labelled 1–16 were spaced equally around its perimeter. A circle with a diameter of 2 m was marked out in the centre of the arena with the letters A–H spaced equally around its perimeter and I, J in the centre.” and “Individuals were asked to stand on a letter (A–J)”. “To avoid any bias due to the initial direction of locomotion, the initial orientation of each individual is randomly facing a number from the outer circle”. A common instruction was given to all the participants: “when we tell you to begin you should start walking at a normal speed and do not stop before being told to do so. You can walk anywhere inside or outside the circle but you have to stay within an arm's length of another individual and you should not talk or gesture to each other.” In addition to the common instructions, informed and uninformed individuals were created by giving the following instructions respectively: to informed individuals, “Go to number X, without leaving the group”; to uninformed individuals, “stay with the group”. The experiment would be ended when the group reached the periphery (the outer circle in Figure 1).

The same instructions were applied to the second experiment (large group with 200 people). The experiment venue is shown in Figure 1b. Informed people were randomly distributed in the inner circle (with a diameter of 12 m).

In order to reproduce the Dyer's experiments, a simulation environment is implemented based on the crowd model described earlier. The results generated from the simulation are compared and analysed in the following sections.

### Simulation Configuration

Based on the above experiment's instructions, the following behaviour rules and modelling configurations for the simulation have been established and are listed in Table 1:

**Table 1:** Experiment instructions and correspond behaviours rules.

Experiment Instructions	Behaviour Rules or Modelling Configurations	Behaviour Type
Walk at a normal speed	Default walking speed applied	Passive
You can go anywhere	Wandering	Active
Go to number X	Seek to (number X)	Active
Without leaving (Stay with) the group	Keep in group	Active
Stay within an arm's length of another individual	Keep certain distance from others	Active
Do not talk or gesture to each other	No information exchange	Passive
Randomly face a number from outer circle	Initial orientation facing a random number	Passive
Avoid collision (no explicit description)	Repulsive effect from crowd	Passive

The passive behaviours are applied to both types of agents all the time in the simulations. The active behaviours are selected by the agent depends on its type (informed individuals or uninformed individuals) and its status (see Figure 2).

In the simulation, the **Passive** behaviours are applied to both types of agents all the time in the simulations. The **Active** behaviours are decided by the agent based on its type and status.

The agent's behaviours are being updated continuously during the simulation. In each update interval (1/60 second), the flowchart (Figure 2) shows how an agent decides its behaviours during the update interval.

The following section provides detailed descriptions on how the instructions have been translated and configured in the simulation:

- Default walking speed

The participants were instructed to walk at normal speed. Dyer et al. mentioned a normal walking speed in the experiments but did not provide an exact value. In our simulation, 0.4±10% m/s was used as the default walking speed. This value was chosen by considering the crowd density in the experiments and assuming the case of normal condition based on Sakuma et al.'s [36] research.

In the core formula (Formula 1), E_s_ represents the contribution of an agent's default walking speed to the behaviour effect. It is determined by an agent's speed *s*, unit scale *u* in the simulation environment (1 pixel : 0.05 metre) and the update frame rate *r* (60 fps in this simulation) of the simulation graphic engine. Therefore, E_s_ is calculated as follows (applied to all behaviour rules in this simulation):
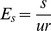
(8)


Formula 8: Conversion of agent's speed to E_s_. *s* is the default walking speed of the agent. The unit of *Default speed* is m/s. *u* is the scale in the simulation. *r is the update frame rate*.

- Wandering

The instruction for uninformed individuals “you can go anywhere” indicates the participants can move freely during the experiment. The “Wandering” behaviour rule in the model can be used to represent this instruction (The parameter F_a_ is set to 1 in this simulation).

- Seek to (number X)

The instruction for informed individuals “go to number X” informs the participants to move to a target position. This can be directly linked to the “Seek to” behaviour in the model which enables an agent to walk directly towards the position of number X. In the formula, F_a_ and F_t_ are set to 1.

- Keep in group

The instruction “Without leaving (Stay with) the group” has been given to all the participants, which is represented by the behaviour rule “keep in group” in this simulation (F_a_ is set to 1 and the group range is set to 5 meters).

- Keep certain distance from others

The instruction “stay within an arm's length of another individual” does not produce any behaviour but serves as a threshold that triggers the behaviour “Keep in group”. Once an agent is out of range with others, it will perform the “keep in group” behaviour to return to the group. Otherwise, it will carry on its default behaviour.

- No information exchange

“In order to minimize the effect of active information transformation, communications between participants are not allowed”, which indicates the participants should follow the original instructions they received to conduct their behaviour during the experiment. Because there is no implicit communication in the simulation, so no special configuration is required.

- Initial orientation facing a random number

The orientations of all the individuals in the simulation are randomly chosen to facing the 16 numbers as it was described in Dyer et al.'s experiment.

- Repulsive effect from others

The repulsive effect helps the individuals adjust their positions while walking and avoid collisions. Although this behaviour cannot be found from the experiment's instructions explicitly, it can be treated as the subconscious behaviour of the participants because it is reasonable for them to stay out of collision when they were told to walk normally. The distance begins to feel repulsive effect is set to 0.7 metre (stay within an arm's length).

## Results and Analysis

### Simulations of Small Group

In Dyer et al.'s small group experiments, 15 groups were tested in four treatments. Informed positions for each treatment are listed as follows in Table 2:

**Table 2:** Starting position of the informed individuals.

Treatment	1	2	3	4
Informed positions	J&E	B&F	C&D	I&J
Treatment description	Core and Periphery	Close Periphery	Far Periphery	2 Cores

Letters refer to the positions that are illustrated in Figure 1a.

In our simulation, each treatment has been run 1600 times (for one treatment, each target number (1–16) was simulated 100 times). The simulation's results were processed through Microsoft Excel 2007 Analysis ToolPak® to generate the statistical and graphical reports.

#### Overall result

**Figure 3:**
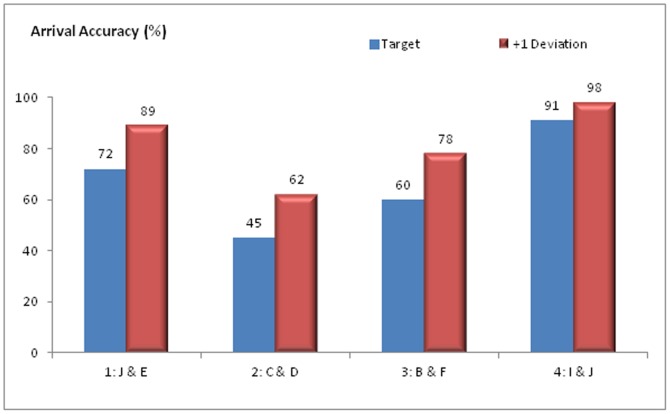
Arrival accuracy for the four treatments. Arrive at the target means the group reached the selected target number (see Figure 1(a) for the arena) first in a simulation. Arrive at +1 deviation means arriving at the number next to the target is also count as arrived at the target.

**Arrival Accuracy and Arrival Time:** Figure 3 shows the arrival accuracy for the four treatments. The order of the arrival accuracy (from high to low) of the treatment is: 4>1>3>2. In addition, the arrival accuracy remains the same order for the four treatments in the case of +1 deviation (arriving at the number next to the target is also counted as arrived at the target).

**Figure 4:**
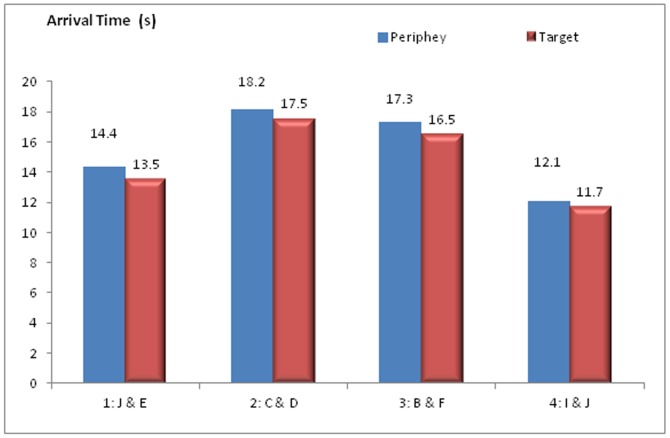
Arrival time - the time taken for the group to reach any number (see **Figure 1(a)** for the arena). Periphery means reaching any number on outer circle includes target.

Figure 4 presents the arrival time of the four treatments. It reveals that the order of the arrival time (from short to long) for the four treatments is: 4>1>3>2, which has the same order of arrival accuracy. Another finding is that the group takes less time to reach the target than to reach the periphery on average, which can be seen in all the four treatments.

The results suggest (by comparing Figure 3 and Figure 4) that the group with higher accuracy level can also reach the target quicker. In addition, the informed individuals could hold back the group if the group is not heading to the right target, which slows down the group movement. This effect is observed in Figure 4 (the group takes longer time to reach Periphery than target)

**Comparison to Dyer et al.'s findings:** Dyer et al.'s experiment indicated treatment 1 (Core and Periphery position, median time about 14 s) and treatment 4 (core position, median time about 21 s) spent less time to arrive the periphery than treatment 3 (B&F, median time about 24 s) but could not determine statistically a difference between other treatments based on its experiment samples (15 groups). The original experiment found that treatment 1 (J&E) has much less deviation on arriving at the target than all the other three treatments, However, the small sample size limited Dyer et al. to analyse the data further.

Our simulation results indicate that when the informed individuals are located at the core positions, the group has better accuracy and less arrival time. It suggests the informed individuals can influence the group more when they are starting at core positions than at peripheral positions. This finding is consistent with Dyer et al.'s experiment findings and are also supported by Leca's [7] research results.

There is one difference between the author's simulation results and Dyer et al.'s experiment results. In our simulations, treatment 4 has better accuracy and arrival time than treatment 1. The reason could be “informed individual in the core position was constrained on mobility and needed some time to find the target while the peripheral positions are easier to move and align with the target” [9] was not considered in our simulation. Due to the informed individual being designed to know the target position from the beginning and having no specific constraint rule applied to the core position, the constraints of the core position on target seeking and movement have not been explicitly modelled in simulation.

#### Detailed results

In our simulation, large amount of simulation data (1600 simulations) has been generated which will enable further analysis on the group behaviour in addition to the original Dyer's analysis.

**Figure 5:**
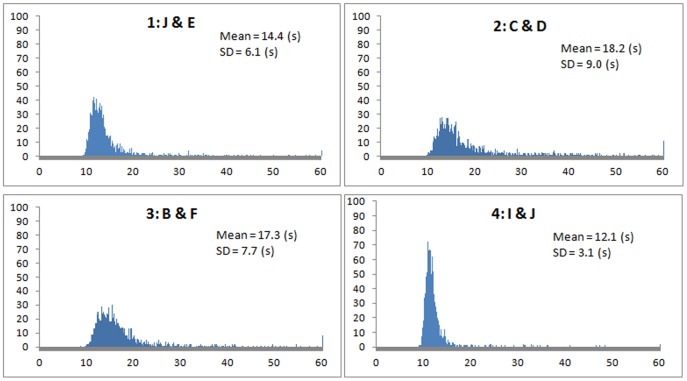
Histogram of arrival time (in 0.1 second interval) to periphery. Y-axis represents the frequency. (Only shows the records that are less than 60 s for better visibility. Within 60 seconds, it contains 99.75%, 99.31%, 99,50%, 99.88% of the 1600 records for each treatment respectively). Histogram was generated by using Excel 2007.

**Comparison of arrival times:** The histograms (Figure 5) show the distribution of arrival times. Treatment 2 and treatment 3 have a quite large SD (standard deviation). The reason could be when considering the size of experiment venue, the distance between the starting positions of informed individuals and target numbers cannot be ignored. This explains why treatment 4 has the narrowest distribution and treatment 2 has the widest. For position I&J (treatment 4), the distances to all the target numbers are the same and as a result, it has the smallest SD. For position C & D (treatment 2), the distances have the most significant variance from the informed positions to target numbers among the four treatments.

**Figure 6:**
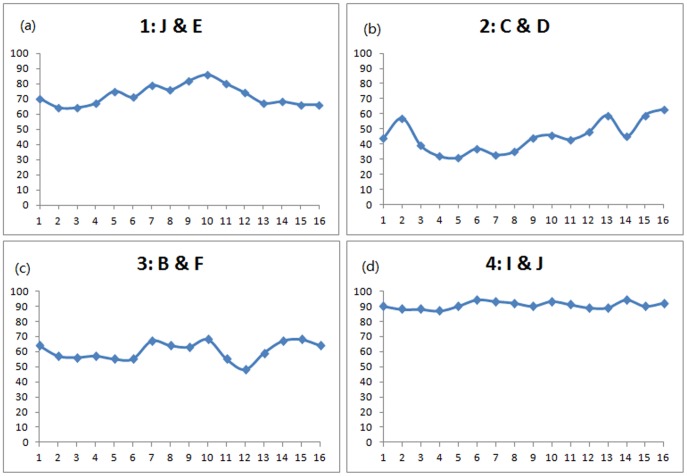
Detailed arrival accuracy for each treatment. X-axis represents the target number in simulation for each treatment. Y-axis represents the accuracy of reaching that target number in simulation.

**Detailed arrival accuracy for each treatment:** Figure 6 shows the group arrival accuracy to target number is also influenced by the positions of informed individuals. It is not surprising to see there is not much difference between the arrival accuracy of target numbers in treatment 4 because the two informed individuals started at the core positions. Treatment 2 has the lowest accuracy level because the two informed individuals were located at the same side of the group next to each other, which reduces their influence to the whole group [7], [9].

**Figure 7:**
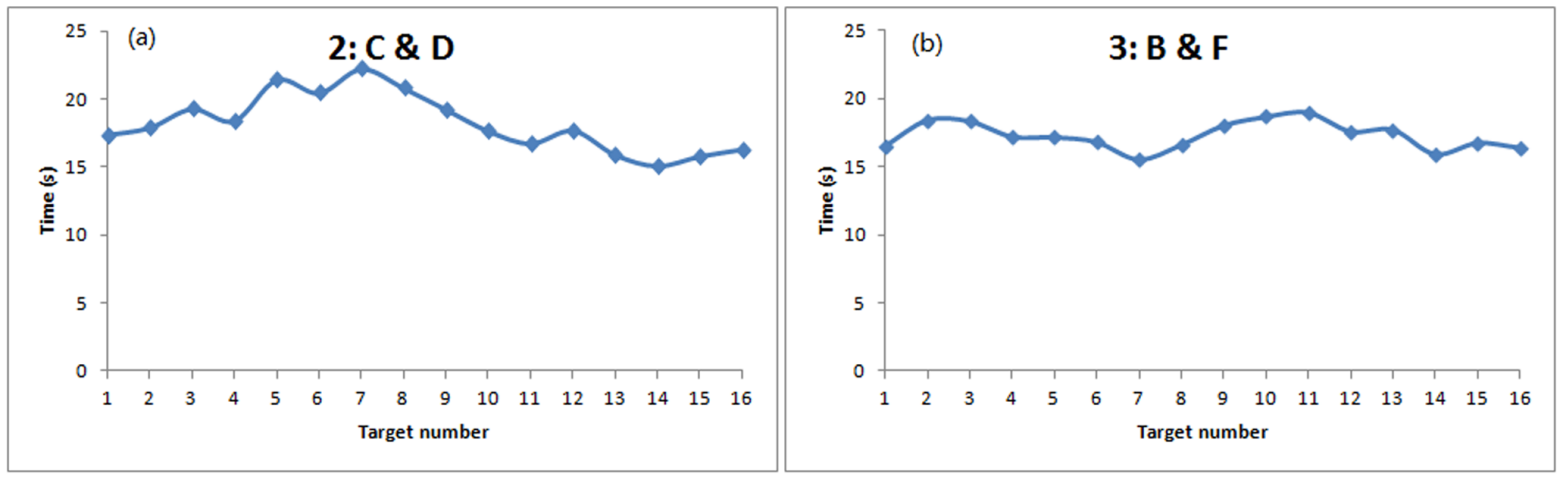
Arrival time to periphery for each target number.

**Figure 8:**
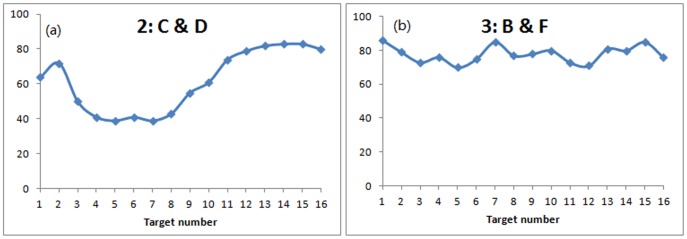
Arrival accuracy for each target number (+1 deviation).

**Influence of peripheral informed people to arrival time and accuracy**: By continuing the analysis on the cases with only peripheral informed individuals (Figure 7 and Figure 8), it indicates when the informed individuals are located on the periphery of the group, it is more difficult for them to guide the group to the target number that are close to them.

Figure 7(a) shows in treatment 2, the group takes more time to reach the periphery for the target number 5, 6, 7, 8 which are actually more close to the starting positions (C&D) of the informed persons. A similar situation has also been found in treatment 3 (Figure 7b): time to arrive at targets number 2, 3, 10, 11 are slightly longer than others.

In terms of arrival accuracy, Figure 8 indicates the peripheral informed individuals also has a negative influence on arrival accuracy. Similar to the arrival time, when the target numbers are close to the informed individuals (target number 4, 5, 6, 7, 8 for treatment 2: C&D and target 2, 3, 4, 10, 11, 12 for treatment 3: B&F), the accuracy level to reach the target are lower than the other targets.

On the other hand, Figure 8 provides another piece of evidence of the positive relationship between the higher accuracy and shorter arrival time.

Although peripheral informed positions show a negative influence on arrival time and accuracy, it should be pointed out that this negative influence only exists when there is no informed individual at the core position. If there is an informed individual at the core position, the other informed individual at the periphery can increase the group arrival accuracy when the target is close to the informed individual at the periphery. It can be seen in Figure 6a, for treatment 1, with one informed individual in the core position J, the peripheral informed position E shows a positive influence. The arrival accuracy actually increases around target number 10. This indicates the initial informed individual at the core position is crucial to the group behaviour.

#### The relationship between speed and accuracy

Franks et al.'s [37] study indicated the trade-off between accuracy and speed (the group sacrificed accuracy to reach a quicker consensus decision) in ant colonies in order to locate a better nest. In another similar experiment (Dyer et al.'s [8]), no such relationship was found between arrival time and accuracy. They considered the reason could be due to the small sample size or the limitation of the experiment settings.

**Figure 9:**
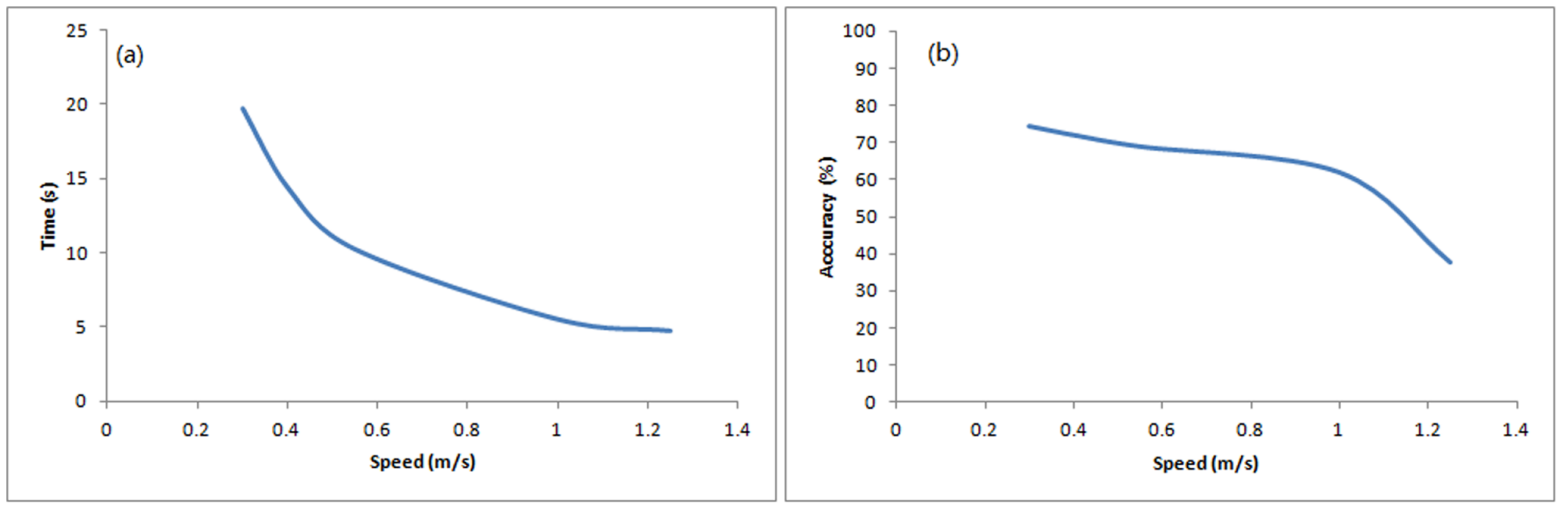
Simulation with various speeds (a) Arrival time; (b) Arrival accuracy. Curves are generated by using Excel 2007.

In order to investigate the relationship between speed and accuracy further, we repeated treatment 1 (J&E) with various speeds (0.3, 0.4, 0.55, 0.8, 1.0 and 1.25 m/s), 1600 times each. The results (Figure 9a) show the arrival time decreases while the walking speed increases. The accuracy trade-off with walking speeds has also been observed (Figure 9b). As a result, it was concluded that quicker arrival time is linked to lower arrival accuracy and such trade-off relationship existed in our simulation. In addition, the arrival time changes more significantly at lower speeds (below 0.5 m/s) than at higher speeds (above 1 m/s), but the accuracy level drops more significantly at high speeds.

### Simulations of Large Group

Dyer et al. concluded the findings on a large group (see Table 3) as anecdotal because only one group of 200 was used to test the experiment:

**Table 3:** Dyer et al.'s anecdotal finds of large group.

Informed Percentage	Arrival Time (second)	Arrival Status
2.5%	222	5% of the group arrived
5%	250	89% of the group arrived
10%	75	100% of the group arrived

In our simulation, the same modelling configurations were used to simulate the experiments of large groups. In order to investigate the relationship between speed, arrival time and numbers of informed people, we have tested the walking speeds at 0.4 m/s, 0.8 m/s and 1.2 m/s with an informed percentage of 5%, 10% and 15% respectively, thus nine treatments together. For each treatment, the simulation has been repeated 100 times.

#### Arrival time and accuracy change with informed percentage

**Figure 10:**
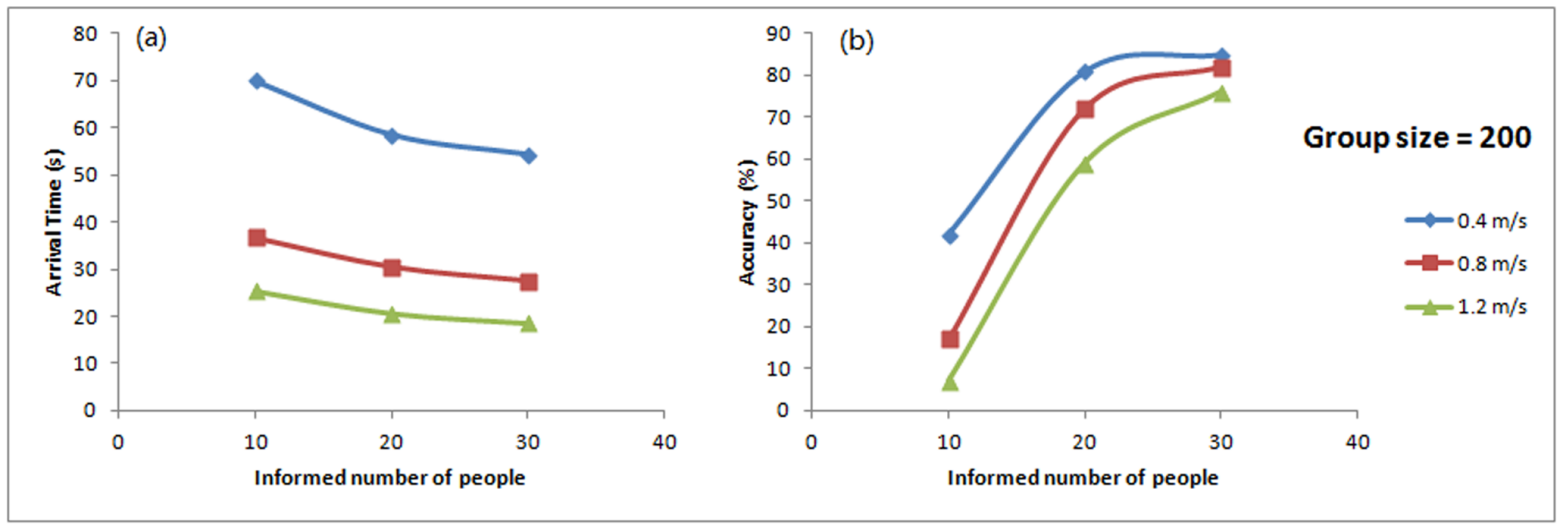
Arrival time and accuracy changed with various informed number of people. The informed percentages are 5%, 10%, and 15% to the numbers of 10, 20, and 30.

Figure 10(a) shows the arrival time has an approximate linear relationship with the number of informed people. Figure 10(b) shows the amount of informed individuals could increase the accuracy of reaching the target and the high accuracy could be reached when the informed individuals are more than 20 (above 10%). This result is consistent with Dyer et al.'s experiment and also supported by Couzin et al.'s [10] simulations in animal group and Pelechano and Balder's [31] findings on the leaders' behaviour in evacuation.

#### Positions of informed individuals in the group

In the simulation, it was observed that the informed individuals' relative positions in the group will gradually move to the edge of the group in the direction of the target. This looks like the informed individuals are leading the group to the direction of the target although they have not been given such instructions/rules. The same behavioural patterns of informed individuals has been reported in Dyer et al.'s [8] other experiments on small groups. Our simulations show that such movements of informed individuals are more evident with large groups, because the place is larger and so the distance to target is longer, which provides more space and time to form such behaviour.

#### Effect of walking speed

**Figure 11:**
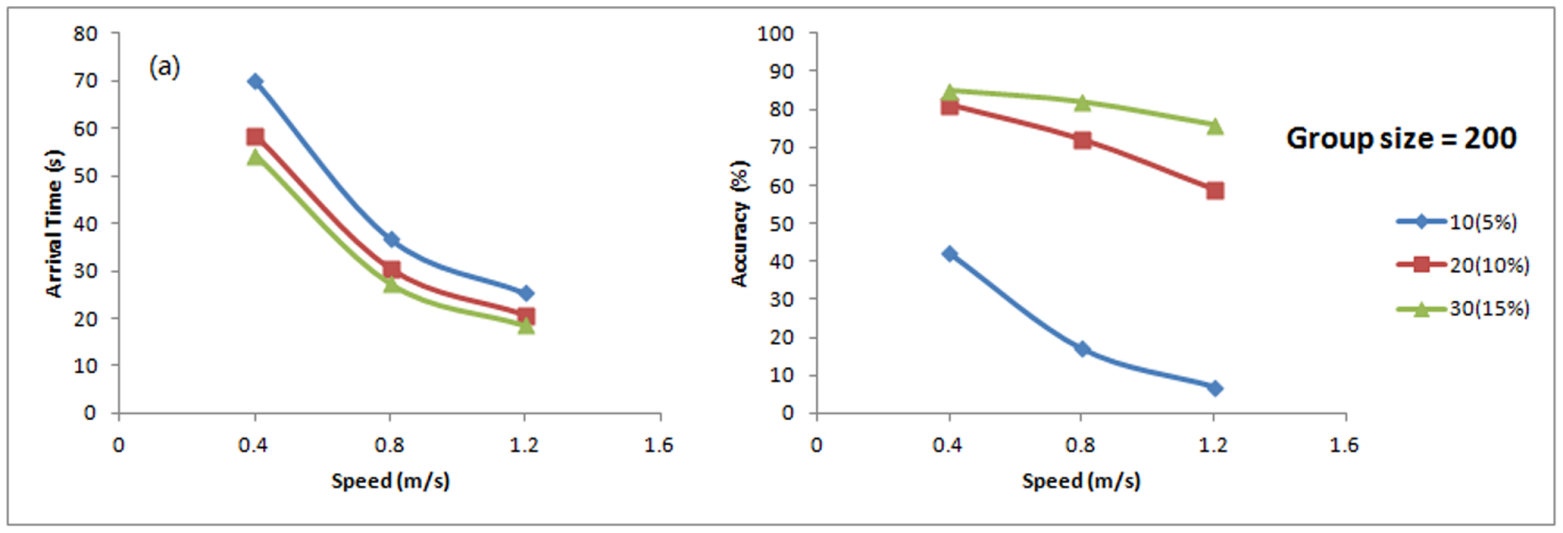
Arrival time (a) and accuracy (b) with various walking speeds.

The simulation's results also reveal how the walking speed could affect the behaviour of the large group, which has been observed in the small groups. Figure 11a shows the arrival time decreases with the increase of speed. Compared to Figure 11a, it can be found that the relationship between arrival time and walking speed in a large group is similar to a small group, which has an inverse relation.

Figure 11b indicates the arrival accuracy and walking speed also have the inverse relation. Furthermore, with a larger percentage of informed individuals, the negative effect of increasing speed becomes less significant. Furthermore, by comparing Figure 9b and Figure 11b, it can be found that the negative effect on accuracy is much less in large group than the small group when speed increases. In the case of a small group (equal to 20% informed), the accuracy dropped from 75% to 45% when the walking speed increased from 0.4 m/s to 1.2 m/s (Figure 9b). In the case of having 15% informed people in large group, the accuracy only changed from 85% to 80% (Figure 11b).

## Conclusion

This paper demonstrated a computer simulation method to study human consensus decision making and leadership. This study has successfully reproduced the Dyer et al.'s experiments in a virtual crowd simulation environment. The simulation results were either consistent with the original experiments results or supported by other similar research. Furthermore, the unified core formula of the crowd model, which can be configured to present different behaviours together with the integration of agent model for high-level decision making, provides the flexibility and accessibility for researchers when studying crowd's behaviours and movements in various scenarios.

In summary, the significance of this study lies in three aspects:

1. Firstly, it validates of the authors' crowd model by comparing with the real-life experiments.
2. Secondly, it provides a simulation environment to study crowd behaviours and additional findings could be discovered in such environment.
3. Finally, this study demonstrates an approach of configuring a generic crowd model into a specific scenario which has not been seen in existing studies.

The additional findings discovered in the simulation during this study are concluded as follows:

1. The phenomena of sacrificing accuracy to reach a quicker consensus decision found in ants colonies [37] has also been discovered in our simulation in both large and small group simulation, which further demonstrates the reliability of the authors' model.
2. The ability of reaching consensus in groups has a direct impact on the time and accuracy of arriving at the target. The earlier the groups can reach a consensus, the quicker and more accurate they arrive at the target. Therefore, the more information or training can be provided to the individual in the crowd, the more effective crowd movement can be controlled.
3. Our simulation indicated that the informed individual at the core position can produce the most positive effect on arrival accuracy and it can also improve the effectiveness of the informed individuals at the peripheral position. It also suggested that if all informed individuals were at the peripheral position, it would take longer time for the group to reach the targets as they are originally closer to them. Therefore, the position of the informed individuals or leaders in the crowd could have significant impact on the crowd movement. Particularly, in emergency simulation, where the deployment of emergency service personnel could be vital to evacuate a large crowd efficiently.
4. Our simulation also confirmed Dyer et al.'s anecdotal evidences on the proportion of leaders required to direct a large crowd. This finding could help determine the number of trained/informed personnel required in an event involving large crowd (such as football match, outdoor concert etc).

The authors also recognise that there were several technical limitations in the simulation:

1. The position of the target number is known to the agent at the beginning of each simulation. Therefore, the process of finding and aligning with the target number for the agent is not considered in the simulation.
2. The group range is defined at 100 pixels (5 metres) for the “keep in group” behaviour in the simulation while different ranges could potentially affect the group behaviour.

## Supporting Information

Code S1 **Pseudo-code for the behaviour effect calculation.**
(DOCX)

Data S1 **Raw data for the ms.**
(ZIP)

Equation S1 **MathType 3.0.**
(ZIP)
