# Supplementary material for: Computer Simulation of Leadership, Consensus Decision Making and Collective Behaviour in Humans
Source: PLoS One. 2014 Jan 17;9(1):e80680. doi: 10.1371/journal.pone.0080680 (PMC3894931; doi:10.1371/journal.pone.0080680)
Supplement: Code S1 — Pseudo-code for the behaviour effect calculation. (DOCX) [file pone.0080680.s001.docx]

- Implementation of “Seek to”

If target exist

If has reached the target

Stop moving

Else

Change the orientation to face the target

Calculate the base movement distance from the default walking speed (E_s_)

Determine the coefficient of the Self Factor (F_a_)

Determine the coefficient of the Target Factor (F_t_)

Update the movement distance by applying the above coefficients

Move forward with the updated distance

Else

Do nothing

- Wandering

Generate a random number in the range of [0, 100]

If the generated number < 5

Generate a random angle in the range of [-18°, +18°]

Turn the current orientation at the randomly generated angle

Else

Calculate the base movement distance from the default walking speed

Determine the coefficient of the Self Factor (F_a_)

Update the movement distance by applying the above coefficients

Move forward with the updated distance

- Repulsive Effect from Crowd

Set the range of the group

Identify all the agents inside the group

For each agent (exclude self) in the group

Calculate the distance to that agent

If distance <= the minimum distance

Set the coefficient of Distance Factor to 1

Else

Set the coefficient of Distance Factor to 1/distance

Change the orientation to back face the agent

Calculate the base movement distance from the default walking speed (E_s_)

Determine the coefficient of the Self Factor (F_a_)

Determine the coefficient of the Target Factor (F_t_)

Update the movement distance by applying the above coefficients

Move forward with the updated distance

End Loop

- Follow the Group

Set the range of the group

Identify all the agents inside the group

Apply behaviour rule “Walk toward the Group”

Apply behaviour rule “Align Direction with Group”

- Walk toward the Group

Set the range of the group

Identify all the agents inside the group

Calculate the average position of all the agents (including self) in the group

Change the orientation to face the average position

Calculate the base movement distance from the default walking speed (E_s_)

Determine the coefficient of the Self Factor (F_a_)

Update the movement distance by applying the above coefficient

Move forward with the updated distance

- Align Direction with Group

Set the range of the group

Identify all the agents inside the group

Calculate the average orientation of all the agents (including self) in the group

Change the orientation to that average orientation

Calculate the base movement distance from the default walking speed (E_s_)

Determine the coefficient of the Self Factor (F_a_)

Update the movement distance by applying the above coefficient

Move forward with the updated distance
